# Development and Characterization of a Three-Dimensional Organotypic In Vitro Oral Cancer Model with Four Co-Cultured Cell Types, Including Patient-Derived Cancer-Associated Fibroblasts

**DOI:** 10.3390/biomedicines12102373

**Published:** 2024-10-17

**Authors:** Yuka Aizawa, Kenta Haga, Nagako Yoshiba, Witsanu Yortchan, Sho Takada, Rintaro Tanaka, Eriko Naito, Tatsuya Abé, Satoshi Maruyama, Manabu Yamazaki, Jun-ichi Tanuma, Kazuyo Igawa, Kei Tomihara, Shinsaku Togo, Kenji Izumi

**Affiliations:** 1 Division of Biomimetics, Faculty of Dentistry & Graduate School of Medical and Dental Sciences, Niigata University, Niigata 951-8514, Japan; aizaway@dent.niigata-u.ac.jp (Y.A.); witsanu.yortchan.wy@niigata-u.ac.jp (W.Y.); takada@dent.niigata-u.ac.jp (S.T.);; 2 Division of Oral and Maxillofacial Surgery, Faculty of Dentistry & Graduate School of Medical and Dental Sciences, Niigata University, Niigata 951-8514, Japan; n-eriko@dent.niigata-u.ac.jp (E.N.);; 3 Division of Reconstructive Surgery for Oral and Maxillofacial Region, Faculty of Dentistry & Graduate School of Medical and Dental Sciences, Niigata University, Niigata 951-8514, Japan; 4 Department of Oral Health and Welfare, Faculty of Dentistry & Graduate School of Medical and Dental Sciences, Niigata University, Niigata 951-8514, Japan; 5 Division of Oral Pathology, Faculty of Dentistry & Graduate School of Medical and Dental Sciences, Niigata University, Niigata 951-8514, Japantanuma@dent.niigata-u.ac.jp (J.-i.T.); 6 Neutron Therapy Research Center, Okayama University, Okayama 700-8558, Japan; igawakazuyo@okayama-u.ac.jp; 7 Department of Respiratory Medicine, Graduate School of Medicine, Juntendo University, Tokyo 113-8421, Japan; shinsaku@juntendo.ac.jp

**Keywords:** oral cancer, cancer-associated fibroblasts, oral mucosa, patient-derived, organotypic culture, 3D in vitro model, polarity

## Abstract

**Background/Objectives:** Cancer organoids have emerged as a valuable tool of three-dimensional (3D) cell cultures to investigate tumor heterogeneity and predict tumor behavior and treatment response. We developed a 3D organotypic culture model of oral squamous cell carcinoma (OSCC) to recapitulate the tumor–stromal interface by co-culturing four cell types, including patient-derived cancer-associated fibroblasts (PD-CAFs). **Methods**: A stainless-steel ring was used twice to create the horizontal positioning of the cancer stroma (adjoining normal oral mucosa connective tissue) and the OSCC layer (surrounding normal oral mucosa epithelial layer). Combined with a structured bi-layered model of the epithelial component and the underlying stroma, this protocol enabled us to construct four distinct portions mimicking the oral cancer tissue arising in the oral mucosa. **Results**: In this model, α-smooth muscle actin-positive PD-CAFs were localized in close proximity to the OSCC layer, suggesting a crosstalk between them. Furthermore, a linear laminin-γ2 expression was lacking at the interface between the OSCC layer and the underlying stromal layer, indicating the loss of the basement membrane-like structure. **Conclusions**: Since the specific 3D architecture and polarity mimicking oral cancer in vivo provides a more accurate milieu of the tumor microenvironment (TME), it could be crucial in elucidating oral cancer TME.

## 1. Introduction

The complex cellular configuration in human tissues is unlikely to be recapitulated in traditional two-dimensional (2D) cultures, which usually result in monolayer cell cultures [1]. In a monolayer, cells exhibit a planar morphology and only interact on the lateral surface. Monolayer culture has recently been judged inept at modeling in vivo behavior because it lacks critical cell–cell and cell–matrix interactions in their native microenvironment [2]. Three-dimensional (3D) cell culture techniques have been created to recapitulate the in vivo physiological environment, where the cell morphology, interactions, and tissue-specific architecture more closely resemble those of native tissues [3]. Therefore, 3D in vitro models are becoming increasingly common and indispensable in various cancer studies to investigate the tumor microenvironment (TME), contributing to a better understanding of interactions among cancer stem cells, cancer stroma cells, and the extracellular matrix (ECM) [2,4,5]. Currently, regardless of scaffold-free and scaffold-based techniques, the popular modalities of 3D culture technologies include spheroids, organoids, organ-on-a-chip (microphysiological system), and the use of 3D bio-printing [6,7,8]. Nonetheless, because each method presents advantages and disadvantages, it is crucial to choose an appropriate system for 3D cell culture, depending on the cancer type and the research purpose [9]. For instance, organoids and spheroids have often been used to study cancer drug discoveries to overcome significant challenges in translating promising preclinical drug candidates tested on monolayer cell culture into the clinic [10,11].

Three-dimensional in vitro oral squamous cell carcinoma (OSCC) models have focused on emulating their native counterparts and creating a more pathophysiologically relevant environment in vitro [4]. Studies using organoids have prevailed as a useful approach using in vitro 3D OSCC models [12,13,14]. Although recent reviews disclosed that 3D OSCC models do not exhibit technological trends [4,15], TME characteristics in OSCC that are recognized key factors in determining a therapeutic response have not been fully applied to 3D OSCC model designs [4]. Similarly to other 3D in vitro tumor models mimicking tissue-specific TME, 3D OSCC models must incorporate three major factors, including cancer heterogeneity, 3D tumor-like geometry, and the vascular system [4]. Thus, it is preferable to incorporate those factors within the OSCC model.

Since OSCC arises in the lining oral mucosa and gingival epithelium, it partially presents an air–liquid interface anatomy facing the oral cavity (luminal side). Therefore, the apical surface is in contact with the air/saliva, while the basal part interacts with mesenchymal cells, the ECM, and the vasculature [4]. Three-dimensional organotypic culture, an initial platform wherein an epidermal layer is reconstituted at the air–liquid interface on top of a type I collagen-based matrix containing dermal fibroblasts [16,17], is a powerful tool to recapitulate the tumor–stroma interface in OSCC, similar to esophageal cancer studies [18]. Previously, we modeled the OSCC organotypic culture to investigate the crosstalk between OSCC and cancer-associated fibroblasts for mechanistic study, co-culturing OSCC cell lines and commercially available cancer-associated fibroblasts (CAFs) [19]. Furthermore, we analyzed cellular damages by X-ray irradiation, boron neutron capture therapy, and carbon ion irradiations using 3D OSCC organotypic culture models by co-culturing OSCC cell lines and oral mucosa/dermal fibroblasts embedded in type I collagen stroma. Our analysis revealed that our models can evaluate radiobiological effects through histopathological examinations [20,21,22].

Recently, we have been able to culture patient-derived CAFs (PD-CAFs) from OSCC tissue and patient-derived normal oral fibroblasts (PD-NOFs) from intact oral mucosa tissue adjacent to an oral cancer lesion simultaneously. We attempted to incorporate PD-CAFs and PD-NOFs into our standard protocol, manufacturing 3D in vitro models to promote our platform technique of assembling the 3D OSCC organotypic culture. Using one stainless-steel partition allowed us to co-culture four cell types, including OSCC cell lines and normal oral mucosa keratinocytes (NOKs). In this study, we developed a 3D organotypic culture OSCC model that could contribute to understanding OSCC biology and assessing cellular responses of oral cancer tissues and their surrounding normal oral mucosa tissue to cancer treatments simultaneously. This model recapitulates the OSCC-specific TME because it includes heterogenic cell components involving PD-CAFs and PD-NOFs and mimics OSCC geometry in vivo, including tumor positioning and physiological properties. Therefore, this complex cellular model could enhance the discovery of new and effective oral cancer treatments.

## 2. Materials and Methods

### 2.1. Procurement of Oral Mucosa and Oral Cancer Tissue

The two protocols for obtaining normal oral mucosa and oral cancer tissue samples were approved by the Niigata University Ethical Committee (Approval #2015-5018: Approval #2022-0300). The oral mucosa tissue samples were collected during dento-alveolar surgeries from patients who visited the Department of Oral Surgery at the Niigata University Medical and Dental Hospital. Oral cancer tissue samples were collected from patients who underwent tumor resection at the Department of Oral Surgery, Niigata University Medical and Dental Hospital. All patients gave informed consent with an understanding of the study. Paired oral cancer tissue and normal oral mucosa samples were obtained from the same en bloc resected specimen. This study’s methodologies conformed to the standards set by the Declaration of Helsinki.

### 2.2. Primary Cell Culture of NOKs

Primary human NOKs were harvested from normal oral mucosa tissue. After rinsing with serum-free EpiLife (0.06 mM Ca^2+^) culture medium (Thermo Fisher Scientific, Waltham, MA, USA), tissue samples were transferred into a 0.025% trypsin/EDTA solution (Thermo Fisher Scientific) with 1.5% antibiotic–antifungal mixture (Thermo Fisher Scientific) and immersed for approximately 16 h at room temperature (RT). Samples were then transferred into a 0.0125% trypsin inhibitor solution (Thermo Fisher Scientific), and the epithelial layer was scraped from the underlying connective tissue using a scalpel and seeded into culture dishes at 5.0 × 10^5^ cells/cm^2^ as p0 cells. NOKs were grown under a humidified atmosphere of 5% CO_2_ at 37 °C. The primary NOK culture was serially passaged, according to our previous reports [22]. The culture medium comprised serum-free EpiLife (0.06 mM Ca^2+^) with Human Keratinocyte Growth Supplement (Thermo Fisher Scientific), gentamicin (5.0 μg/mL; Thermo Fisher Scientific), and amphotericin B (0.375 μg/mL; Thermo Fisher Scientific) and was referred to as a complete medium. NOKs were fed every other day. p1–p3 cells were used for further analyses.

### 2.3. OSCC Cell Culture

The human OSCC cell lines (HSC-3 and HSC-4; JCRB) were used. The HSC-3 (RRID: CVCL_1288; JCRB0623) and HSC-4 (RRID: CVCL_1289; JCRB0624) cell lines derived from human tongue squamous cell carcinoma (SCC) were obtained from the JCRB Cell Bank (Ibaraki, Japan). The two cell lines were authenticated by short tandem repeat profiling in June 2024, and all experiments were performed with mycoplasma-free cells (Files S1 and S2). The cells were maintained in Dulbecco’s modified Eagle medium (DMEM; Thermo Fisher Scientific) with 10% fetal bovine serum (FBS; Corning, New York, NY, USA), supplemented with gentamicin (5.0 μg/mL) and amphotericin B (0.375 μg/mL).

### 2.4. Primary PD-CAFs and PD-NOFs Cell Culture

**Figure 1:**
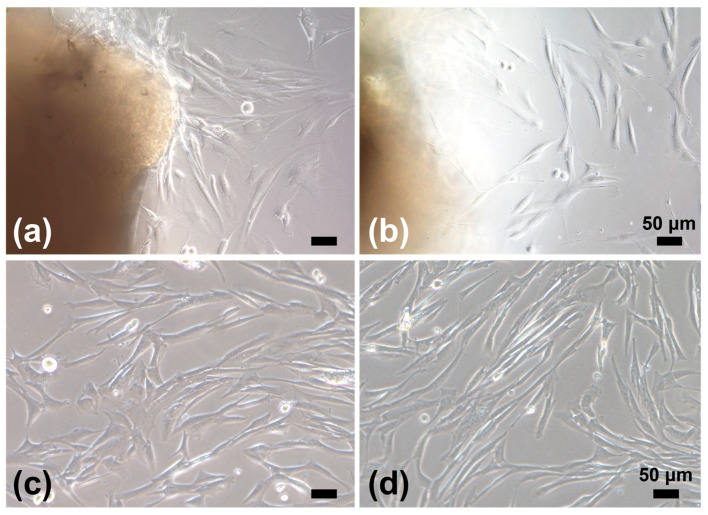
Phase-contrast microscopic images of PD-CAFs (**a**,**c**) and PD-NOFs (**b**,**d**). p0 cells in culture (**a**,**b**). p1 cells in culture (**c**,**d**). Scale bar = 50 μm.

Pieces of tissue samples were trimmed off from the cancer tissue core and the margin of the normal oral mucosa, incised at the resection, to isolate CAFs and NOFs, respectively (Supplementary Figure S1). Tissue samples of approximately 2 × 2 mm were cleaned and minced with a scalpel in DMEM. For primary CAF culture, the cancer specimens were grated onto a 60 mm dish (Corning, NY, USA) using forceps, whose surface was finely scratched with a scalpel, serving as a file (Supplementary Figure S2). This procedure adopted a micro-tissue explant technique. Before the surface dried, DMEM with 10% FBS supplemented with gentamicin (5.0 μg/mL) and amphotericin B (0.375 μg/mL) was gently added. The medium was changed every three days. Within a week, CAFs (referred to as “P0”) were grown into a spindle-shaped fibroblast-like morphology (Figure 1). After CAFs reached approximately 80% confluence, p0 cells were detached with 0.025% trypsin/EDTA (Thermo Fisher Scientific), neutralized with DMEM containing 10% FBS, replated, and grown as p1 cells at 1.3–2.5 × 10^4^ cells/cm^2^. p3–p5 cells were used for further analyses.

Pieces of the normal oral mucosa sample were treated in the same way as NOK cell culture. After the epithelial layer was scraped off, primary NOF culture was established via a tissue explant culture technique from the underlying connective tissue, as previously reported (Figure 1) [22]. NOFs were fed every two days with DMEM containing 10% FBS supplemented with gentamicin (5.0 μg/mL) and amphotericin B (0.375 μg/mL). After the NOF outgrowth from the tissue pieces reached approximately 80% confluence, NOFs were replated and cultured at 1.3–2.5 × 10^4^ cells/cm^2^ in separate culture vessels. NOFs from p3–p5 were used for further analyses. Despite the use of p3–p5 cells, PD-CAFs and PD-NOFs were successfully passaged after p6 in our protocol.

### 2.5. Fabrication of a 3D Organotypic Culture Model

**Figure 2:**
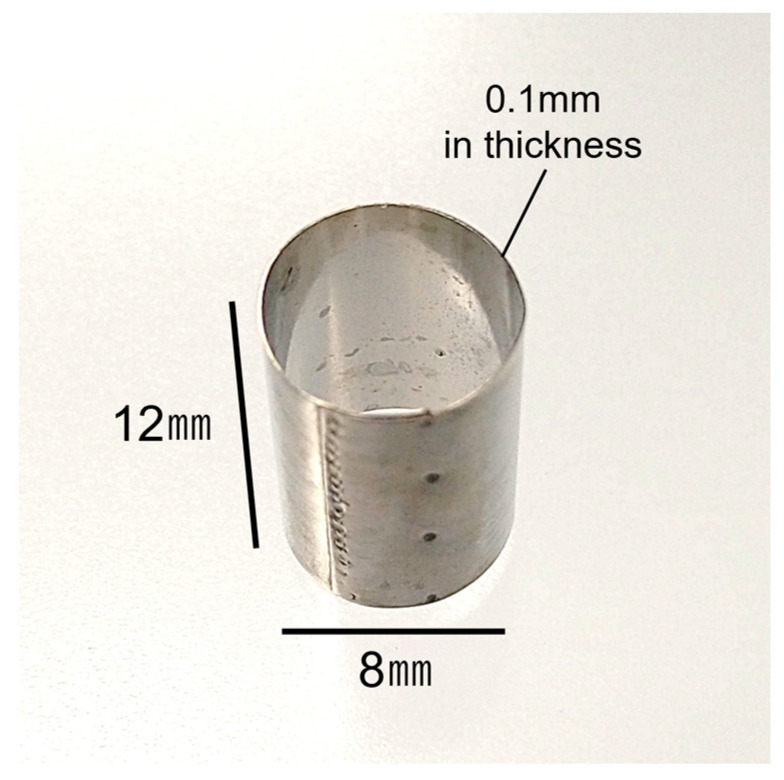
Appearance of a stainless-steel partition used at day 0 during manufacturing.

**Figure 3:**
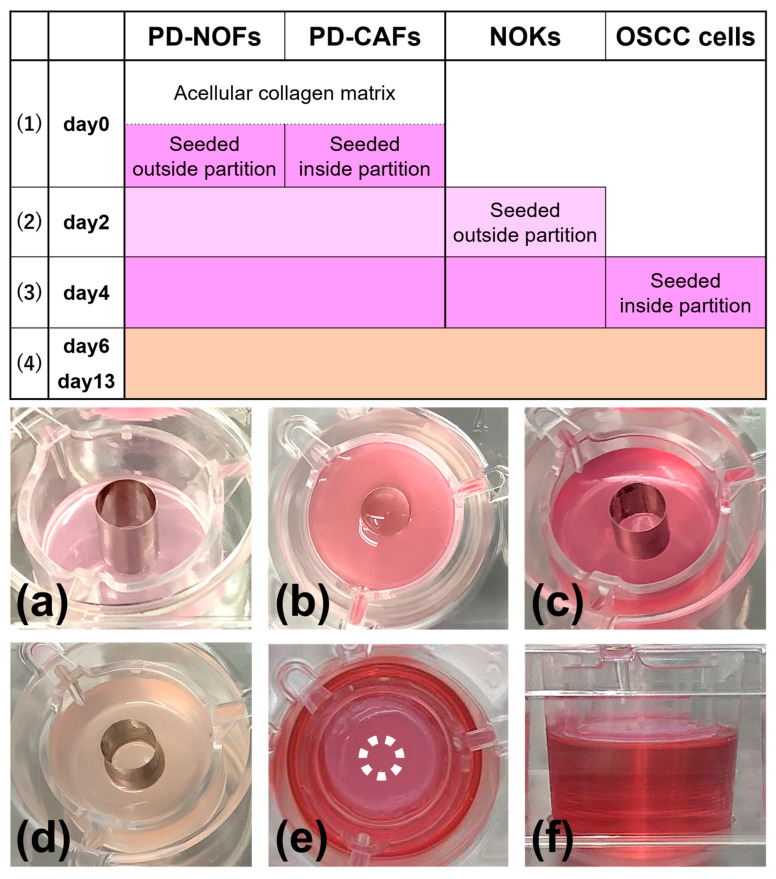
Manufacturing protocol of the 3D organotypic in vitro oral cancer model. The model was cultured in a submerged condition for the first six days (1)–(3), raised at the air–liquid interface on day 6, and cultured for another seven days (4). Depending on the manufacturing procedure, the model was fed with three different culture media: DMEM containing 10% FBS, serum-free EpiLife (0.06 mM Ca^2+^) containing Human Keratinocyte Growth Supplement (complete medium), and a 1:1 mixture of DMEM and complete medium containing 10% FBS. In the manufacturing protocol, three culture media of DMEM containing 10% FBS, complete medium, and a 1:1 mixture of DMEM and complete medium containing 10% FBS are shown in dark pink, light pink, and beige, respectively. Macroscopic appearances of the model are displayed (**a**–**f**). Oblique view at day 0 before seeding PD-CAFs and PD-NOFs (**a**). Top view at day 0 after seeding PD-CAFs and PD-NOFs (**b**). Oblique view on day 2 before seeding NOKs (**c**). Oblique view on day 4 before seeding HSC-3/HSC-4 cells (**d**). Top and side views on day 6 at the air–liquid interface, respectively (**e**,**f**). White dots indicate the partition’s original position (**e**).

The protocol to fabricate a 3D organotypic oral cancer culture model was based on our previous 3D in vitro oral cancer and normal oral mucosa models [19,20,21,22]. Using a stainless-steel partition (Kobayashi Seisakusho Ltd., Niigata, Japan) (Figure 2) allowed us to co-culture four different cells: PD-NOFs, PD-CAFs, NOKs, and OSCC cell lines. The protocol includes four procedures: (1) fabricating a stromal layer, (2) seeding NOKs, (3) seeding HSC-3/HSC-4 cells, and (4) air–liquid interface culture (Figure 3).

(1) (day 0) 1 mL of a cellular type I collagen matrix (Cellmatrix Type I-A, Nitta Gelatin, Osaka, Japan) was prepared according to the manufacturer’s instructions and poured into a tissue-culture insert of a six-well Thincert deep-well plate (Greiner Bio, Kremsmünster, Austria) to gelatinize. A stainless-steel partition, 12 mm high and with an inner diameter of 8 mm, was placed in the center. Subsequently, PD-NOFs (5.5 × 10^5^ cells) mixed with 3300 µL of type I collagen matrix and PD-CAFs (7.5 × 10^4^ cells) mixed with 450 µL of type I collagen matrix were poured into the outside and inside of the partition, respectively. After the plate was incubated for 5 min under a humidified atmosphere of 5% CO_2_ at 37 °C, the partition was slowly removed, and the resulting “stromal layer” was incubated for another 30 min. A quantity of 22 mL of DMEM with 10% FBS was added to each well, resulting in a submerged condition. The stromal layer, including PD-NOFs and PD-CAFs, was cultured for two days.

(2) (day 2) After removing the culture media, a stainless-steel partition of 8.5 mm height was placed on the center of the stromal layer. A cell suspension containing 1.0 × 10^6^ NOKs in 100 µL of the complete medium was seeded onto the stromal layer surface, outside of the stainless-steel partition, and cultured with the complete medium for another two days in a submerged condition. The partition was left as it was.

(3) (day 4) After removing the culture media, the cell suspension containing 5.0 × 10^5^ HSC-3/HSC-4 cells in 50 µL of DMEM with 10% FBS was seeded onto the stromal layer surface inside the partition and cultured with DMEM with 10% FBS for another two days in a submerged condition.

(4) (day 6–13) Two days later, after aspirating the medium, the partition was slowly removed, and the construct was separated from the sidewall of the tissue-culture insert using a 200-μL micropipette tip to allow contraction. Thus, the model, originally 24 mm in diameter, shrank a little. Subsequently, 17 mL of the 1:1 mixture (DMEM and complete medium with 10% FBS) was added outside of the tissue-culture insert, creating an air–liquid interface culture to promote epithelial stratification of normal oral mucosa. The medium was refreshed every two days, and a 3D organotypic oral cancer model was completed on day 13. According to the protocol above, 98 models were manufactured in this study.

### 2.6. Stromal Layer Cell Labeling

Each fibroblast was prelabeled with PKH-26 (PKH-26 Red Fluorescent Cell Linker Kit, Sigma-Aldrich, St. Louis, MO, USA) and PKH-67 (PKH-67 Green Fluorescent Cell Linker Kit, Sigma-Aldrich), according to the manufacturer’s instructions, to analyze the location and distribution of PD-CAFs and PD-NOFs, respectively, in the model’s stromal layer. The organotypic models were fabricated as described above.

### 2.7. Preparation for Histopathological Examinations and Characterization of 3D Model

Three-dimensional models manufactured according to a standard protocol were cut into two pieces. One specimen was fixed in 4% paraformaldehyde (PFA) and embedded in paraffin. Sections of 3.5 μm thickness were prepared for histopathological examination, stained with hematoxylin-eosin (H&E), and immunohistochemistry. The other specimen was embedded in a Tissue-Tek Optimal Cutting Temperature Compound (Sakura Finetek, Tokyo, Japan) and snap-frozen in liquid nitrogen. The frozen sections, cut into 10 μm thick pieces, were fixed in acetone for 10 min at 4 °C for fluorescent immunohistochemistry [23]. The 10-μm thick frozen sections prelabeled with PKH-26 and PKH-67 were fixed in 4% PFA for 3 min at 4 °C to detect CAFs and NOFs in the model’s stromal layer.

### 2.8. Immunofluorescence

CAFs and NOFs were seeded into a four-well chamber slide (Matsunami Glass, Osaka, Japan) at 2.0 × 10^3^ cells/chamber during passaging cells to characterize PD-CAFs and PD-NOFs in a monolayer culture. When the cells reached 70%–80% confluency, they were washed with phosphate-buffered saline (PBS) and fixed with 4% PFA at RT for 15 min. They were permeabilized with 0.2% Triton X-100 in PBS and incubated with 2% normal goat serum (Thermo Fisher Scientific) in PBS at RT for 30 min to block non-specific binding. The cells were immunostained with antibodies for alpha-smooth muscle actin (α-SMA) (dilution 1:100; mouse monoclonal; clone 1A4; Dako, Agilent Technologies, Inc., Santa Clara, CA, USA) and vimentin (dilution 1:200; rabbit monoclonal; clone D21H3; Cell Signaling Technology, Inc., Danvers, MA, USA) at RT for 2 h. After washing with PBS, the cells were reacted with Alexa Fluor™ 488 goat anti-mouse IgG secondary antibody (1:200; cat. no. A11011 Thermo Fisher Scientific) for α-SMA and Alexa Fluor™ 594 goat anti-rabbit IgG secondary antibody (1:200; cat. no. A11012; Thermo Fisher Scientific) at RT for 60 min. Hoechst33342 (Molecular Probes, Eugene, OR, USA) was used to counterstain the cells. The coverslips were mounted with TrueVIEW^®^ Autofluorescence Quenching Kit (Vector Laboratories, Inc., Burlingame, CA, USA). An all-in-one microscope (BZ-X710, KEYENCE, Osaka, Japan) was used to obtain fluorescent images.

### 2.9. Immunohistochemistry

Frozen sections were stained by fluorescent immunohistochemistry to detect the colocalization of laminin-γ2 and integrin-α6 in the model. The primary antibodies and dilutions used were integrin-α6 (GoH3) (1:100, Rat IgG_2_; R&D Systems, Minneapolis, MN, USA) and laminin-γ2 (laminin-5) (P3E4) (1:100, Mouse IgG_1_; Merck Millipore, Burlington, NJ, USA) in PBS. Primary antibody incubation was performed at RT for 2 h. After rinsing with PBS, the primary antibodies were detected using Alexa Fluor 488 goat anti-mouse IgG (1:150; Thermo Fisher Scientific) and Cy3 goat anti-rat IgG (1:450; Jackson ImmunoResearch, Ely, UK) at RT for 2 h. Additionally, the sections were incubated with Alexa Fluor 647 goat anti-mouse IgG (1:150; Thermo Fisher Scientific) to observe the laminin-γ2 expression and the distribution of PKH-26- and PKH-67-prelabeled PD-CAFs/NOFs. The sections were counterstained with ProLOng Diamond Antifade Mountant with DAPI (Thermo Fisher Scientific). All fluorescence microscopy images were obtained with an all-in-one microscope (BZ-X710, KEYENCE) or a Nikon E-800 fluorescent microscope equipped with a digital microscope camera (DP-80, Olympus, Tokyo, Japan). Images were acquired and adjusted with Adobe Photoshop version 25.11.0 (Adobe Systems Inc., San Jose, CA, USA).

Paraffin-embedded sections were immunostained with antibodies for Ki-67 (dilution of 1:50; mouse monoclonal; Santa Cruz Biotechnology, Dallas, TX, USA) as a cell proliferation marker and α-SMA (dilution 1:100) and LRRC15 (dilution 1:125; rabbit polyclonal; MyBioSource, San Diego, CA, USA) as CAF markers to further characterize the model. After deparaffinization, sections were immersed in methanol with 0.3% hydrogen peroxide for 30 min at RT to quench endogenous peroxidase; they were then treated with Tris-EDTA buffer (10 mM Tris Base, 1 mM EDTA solution, 0.05% Tween 20, pH 9.0) in an autoclave at 121 °C for 10 min for antigen retrieval and allowed to cool at RT. Sections were incubated with 5% milk protein in PBS for 30 min to block non-specific protein binding and with primary antibodies at 4 °C overnight. After washing with PBS, Envision Plus (Dako) was applied to the sections at RT for 90 min. Signals were developed with 3,3′-diaminobenzidine (Dako) at RT for up to 30 min depending on the antibodies, and counterstained with hematoxylin.

## 3. Results

### 3.1. Characterization of PD-CAFs and PD-NOFs

#### 3.1.1. Characterization of PD-CAFs

**Figure 4:**
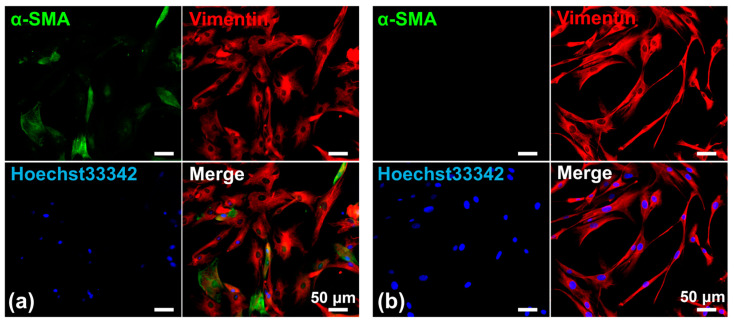
Representative double-immunofluorescent staining of PD-CAFs (**a**) and PD-NOFs (**b**). PD-CAFs and PD-NOFs in a 2D culture were stained against α-SMA (green), vimentin (red), and Hoechst33342 (blue). Scale bar = 50 μm.

PD-CAFs were spindle-shaped and grew in a storiform pattern, very similar to PD-NOFs in 2D culture conditions. Therefore, PD-CAFs were morphologically indistinguishable from NOFs under a phase-contrast microscope. Nonetheless, the cytoplasm of PD-CAFs appeared to be plumper than that of PD-NOFs, indicating the characteristic feature of myofibroblasts [24]. Additionally, we displayed α-SMA expression in PD-CAFs and PD-NOFs under 2D cell culture conditions to determine whether α-SMA could be used as a reliable marker for PD-CAFs. The immunofluorescence finding indicated that α-SMA was expressed in most PD-CAFs but barely in PD-NOFs, although vimentin was expressed in PD-CAFs and PD-NOFs, consistent with our previous report (Figure 4) [19].

#### 3.1.2. Characterization of PD-CAFs in the Surgical Specimen

We also confirmed that α-SMA-positive PD-CAFs were present in the resected OSCC tissue from which the cancer tissue core was harvested (Supplementary Figure S3). α-SMA-positive fibroblasts were not found in the margin of the normal oral mucosa incised, indicating that α-SMA-positive cells grown in our culture system are likely to be PD-CAFs in OSCC lesions.

### 3.2. Histopathological Findings of a 3D Organotypic In Vitro Oral Cancer Model

**Figure 5:**
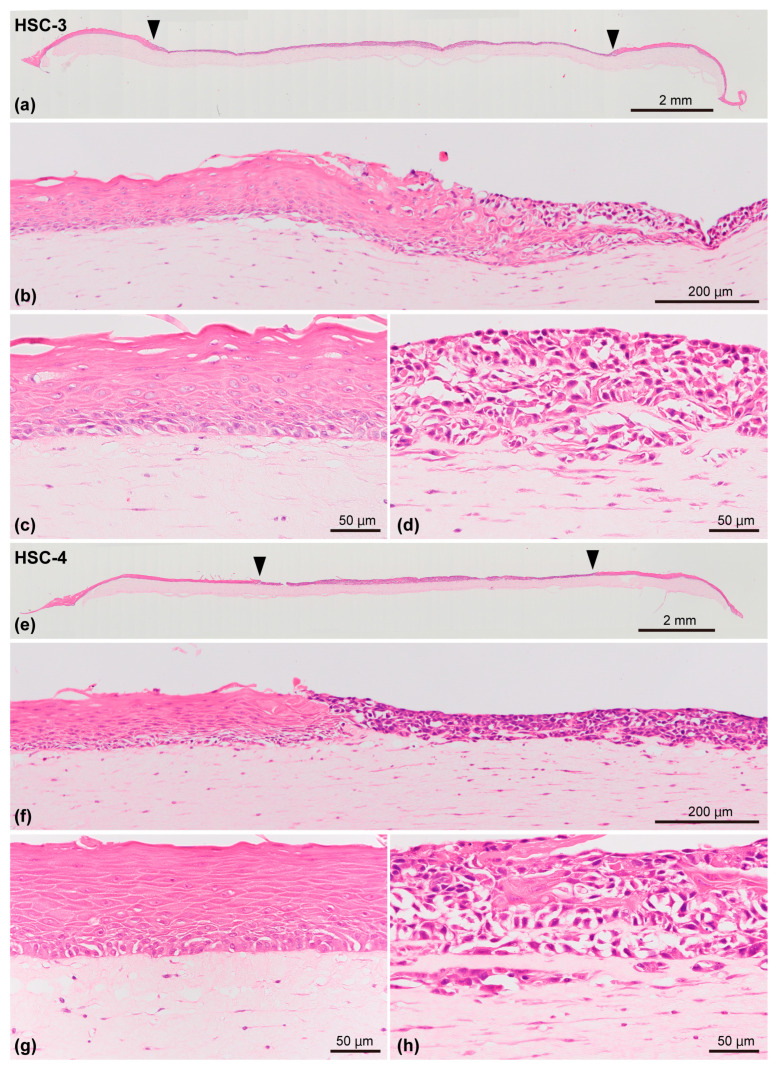
Representative histopathological appearance of the 3D organotypic in vitro model of oral cancer comprising HSC-3 cells (**a**–**d**) and HSC-4 cells (**e**–**h**), respectively, stained with H&E. Entire view of the model with seeded HSC-3 cells (**a**) and HSC-4 cells (**e**) on top of the stromal layer. Two arrowheads indicate the borders between the “normal oral mucosa” and “oral cancer tissue” portions (**a**,**e**). The area between the two arrowheads in the middle indicates the “oral cancer tissue” portion. Magnified microscopic images of the border area between normal oral mucosa and cancer tissue (**b**,**f**), “normal oral mucosa” portion (**c**,**g**), and “oral cancer tissue” portion (**d**,**h**) are also displayed. Scale bar in (**a**,**e**): 2 mm. Scale bar in (**b**,**f**): 200 μm. Scale bar in (**c**,**d**,**g**,**h**): 50 μm.

This model included the stromal layer and overlying epithelial component comprising the OSCC (HSC-3 or HSC-4 cells) layer surrounded by the oral mucosa epithelial layer. The OSCC layer and oral mucosa epithelial layer were located on the stromal layer in which the PD-CAFs or PD-NOFs were initially seeded and resided, respectively. This structure resulted in a 3D organotypic oral cancer model co-cultured with four cell types. In the epithelial component, histopathologically, the OSCC layer was found in the model’s center, and the stratified squamous epithelial layer was juxtaposed to the OSCC layer on both sides (Figure 5a,e). However, the area of OSCC (HSC-3 and HSC-4 cells) layers was larger than initially seeded, as the area of the oral mucosa epithelial layer on both sides decreased. HSC-3 and HSC-4 cells crawled over the oral mucosa epithelial layer at the area where the HSC-3/HSC-4 cells and NOKs were in direct contact (Figure 5b,f). The oral mucosa epithelial layer displayed good keratinization and regular arrangement of basal cells, resulting in a well-organized stratified epithelium (Figure 5c,g). In the OSCC layer, round to oval-shaped HSC-3 cells formed a stratified layer without keratinization, similarly to our previous study [19,22] (Figure 5d). Although the cancer invasion into the underlying stromal layer was not obvious, as indicated in our previous study [19], an irregular border between the HSC-3 cell layer and the underlying cancer stroma was observed, implying the initiation of invasion (Figure 5d). Similarly to HSC-3 cells, HSC-4 cells also developed a well-stratified cancer cell layer (Figure 5h). However, similarly to our previous study [19,22], many keratinized foci were found within the cancer cell layer, indicating well-differentiated carcinoma. Additionally, the border between the OSCC layer and the underlying cancer stroma was similar to that of HSC-3 cells.

Furthermore, the stromal layer in this model was continuous and well-integrated with the overlying epithelial component and was relatively homogeneous based on histopathological examination. Even though PD-CAFs and PD-NOFs were seeded separately and cultured under HSC-3/HSC-4 cells and NOKs for 9 and 11 days, respectively (Figure 5a,e), the differences in cell density and distribution pattern between PD-CAFs and PD-NOFs were hardly detected within the stromal layer in which they were dwelled.

### 3.3. PD-CAF and PD-NOF Distribution in the Stromal Layer

**Figure 6:**
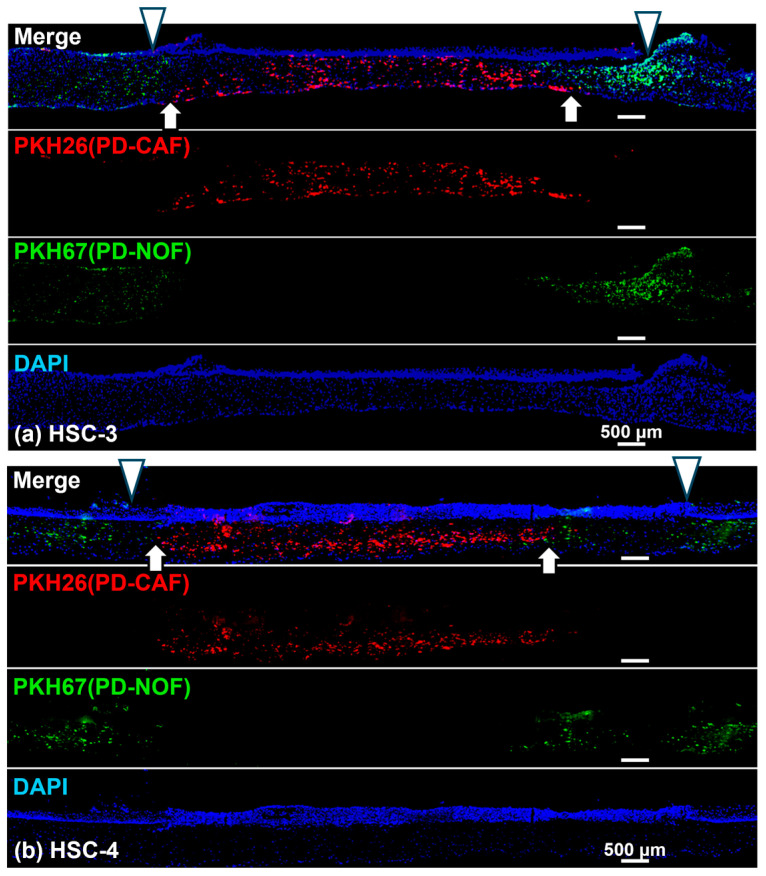
Representative immunohistochemical findings of the 3D organotypic in vitro oral cancer model comprising HSC-3 (**a**) and HSC-4 (**b**) cells, respectively. Entire view of the model stained with DAPI (Blue). PD-CAFs and PD-NOFs populated in the stromal layer were prelabeled with PKH-26 (red) and PKH-67 (green), respectively, before the manufacturing model. Two white arrowheads on the top in the merged images indicate the borders between the “normal oral mucosa” and “oral cancer tissue” portions (**a**,**b**). Two white arrows at the bottom in the merged images indicate the borders between the underlying stromal layer where PD-CAFs and PD-NOFs reside (**a**,**b**). Scale bar = 500 μm.

We prelabeled PD-CAFs and PD-NOFs with PKH-26 and PKH-67, respectively, to determine their distribution in the stromal layer before manufacturing the 3D organotypic models. Consequently, the stromal layer was well-delineated by two portions comprising PD-CAFs and PD-NOFs (Figure 6a,b). This observation resulted from the portion of PKH-26-labeled PD-CAFs being sandwiched by the adjacent portion of PKH-67-labeled PD-NOFs. This finding suggests that most PD-CAFs and PD-NOFs remained at the original seeding site for 13 days. Additionally, at the interface between the two stroma portions, only a few PD-CAFs and PD-NOFs migrated into the adjacent portion, indicating the presence of a thin zone intermingled with those two cells. This resulted in the model’s basic structure maintaining a vertical positional relationship between the “oral cancer tissue” and surrounding “normal oral mucosa” (Figure 6a,b).

However, the gap between the border of cancer stroma and normal oral mucosa connective tissue (white arrow) and the border of OSCC layer and oral mucosa epithelial layer (white arrowhead) confirmed that the HSC-3 and HSC-4 cells expanded outward and overlaid the portion of the normal oral mucosa connective tissue containing PD-NOFs, consistent with the histopathological finding displayed in Figure 5a,b. Therefore, due to the outward expansion, the margin of the OSCC layer was located on the oral mucosa connective tissue with PD-NOFs in this 3D organotypic model.

### 3.4. Characterization of the 3D Organotypic In Vitro Oral Cancer Model

#### 3.4.1. Laminin-γ2 Expression

**Figure 7:**
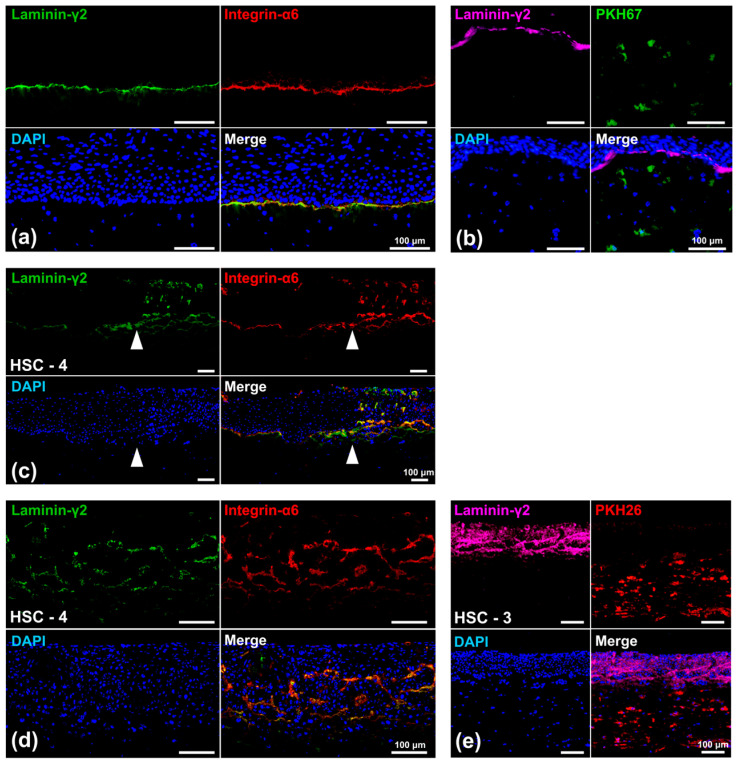
Representative immunohistochemical findings of the 3D organotypic in vitro oral cancer model comprising HSC-3 or HSC-4 cells. Microscopic images of the “normal oral mucosa” portion (**a**,**b**), the border area between normal oral mucosa and cancer tissue (HSC-4 cells) (**c**), and the “oral cancer tissue” portion ((**d**): HSC-4 cells; (**e**): HSC-3 cells) are displayed. White arrowheads indicate the border between NOKs and oral cancer (HSC-4) cells (**c**). Those models were examined with immunofluorescent staining against laminin-γ2 (green), integrin-α6 (red), and DAPI (blue) (**a**,**c**,**d**). A linear co-expression of laminin-γ2 and integrin-α6 was observed at the basement membrane zone in the merged image of the “normal oral mucosa” portion (**a**,**c**). The co-expression of laminin-γ2 and integrin-α6 was observed within the OSCC (HSC-4 cells) layer in the merged image of the “oral cancer tissue” portion (**c**,**d**). Other models, in which PD-NOFs and PD-CAFs were prelabeled with PKH-67 (green) and PKH-26 (red), respectively, were examined by immunofluorescent staining against laminin-γ2 (purple) and DAPI (blue) (**b**,**e**). Scale bar = 100 μm.

We examined the expression pattern of laminin-γ2, a component of the basement membrane, co-localized with integrin-α6, a specific laminin receptor, to further characterize the model. The linear expression of laminin-γ2, co-expressed with integrin-α6, was observed at the basal side of the basal cells in the oral mucosa epithelial layer (Figure 7a). The co-expression pattern was consistent with that of in vivo oral mucosa (Supplementary Figure S4). The co-expression of laminin-γ2 and integrin-α6 in this layer suggested the formation of a basement membrane-like structure at the interface between the oral mucosa epithelial layer and the underlying connective tissue. Moreover, the laminin-γ2 linear expression was not in contact with PKH-67-labeled PD-NOFs (Figure 7b). In contrast, their continuous co-expression abruptly disappeared at the border where the oral mucosa epithelial layer came in contact with the OSCC layer, suggesting a lack of basement membrane-like structure in the OSCC layer (Figure 7c). Within that layer, the HSC-4 cell layer displayed a diffuse laminin-γ2 expression, mostly co-expressed with integrin-α6 (Figure 7d). Additionally, no linear co-expression of integrin-α6 and laminin-γ2 was observed at the interface with the underlying stromal layer. PKH-26-labeled PD-CAFs did not have direct contact with the laminin-γ2 deposition in the OSCC layer, although the diffuse expression was more remarkable in HSC-3 cells than in HSC-4 cells (Figure 7e)

#### 3.4.2. Ki-67, a Proliferation Marker Expression

**Figure 8:**
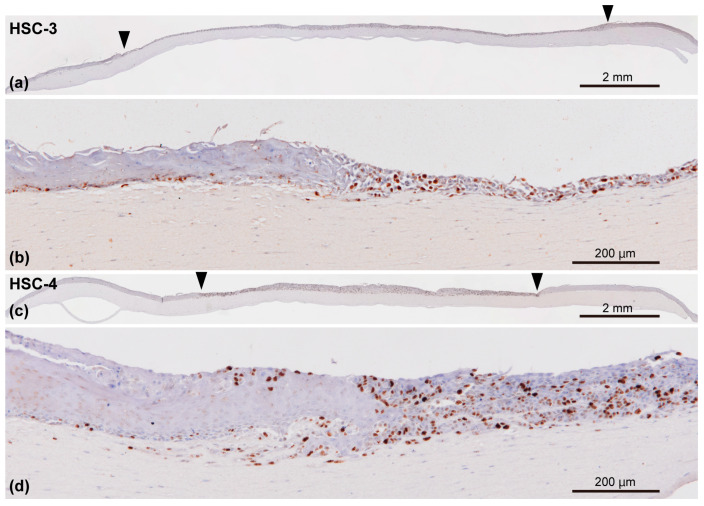
Representative immunohistochemical appearances of the 3D organotypic in vitro oral cancer model comprising HSC-3 (**a**,**b**) and HSC-4 cells (**c**,**d**) examined by immunostaining against Ki-67, a proliferation marker. The specimens were counterstained with hematoxylin. Entire view of the model seeded HSC-3 (**a**) and HSC-4 cells (**c**) on top of the stromal layer. Two arrowheads indicate the borders between the “normal oral mucosa” and “oral cancer tissue” portions (**a**,**c**). Magnified microscopic images of the border area between normal oral mucosa and oral cancer tissue are also presented in (**b**,**d**). Scale bar in (**a**,**c**): 2 mm. Scale bar in (**b**,**d**): 200 μm.

We examined Ki-67 expression, a cell proliferation marker, in the 3D organotypic oral cancer model to determine more characteristics of this model (Figure 8a,c). In the oral mucosa epithelial layer, Ki-67-positive cells were mainly present in the basal layer. Conversely, the Ki-67-positive cells were randomly distributed in the entire OSCC (HSC-3/HSC-4 cells) layer (Figure 8b,d). Although the expression pattern of Ki-67-positive cells was relatively well-delineated between the oral mucosa epithelial layer and OSCC layers (Figure 8b,d), Ki-67-positive cells were also observed underneath and on top of the adjacent normal oral mucosa epithelial layer.

#### 3.4.3. Expression of α-SMA and LRRC15, Potential CAF Markers

**Figure 9:**
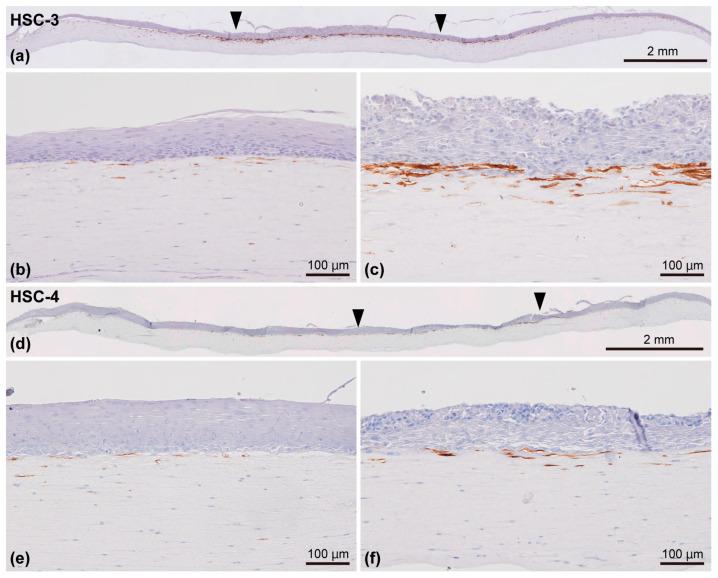
Representative immunohistochemical appearances of the 3D organotypic in vitro oral cancer model comprising HSC-3 (**a**–**c**) and HSC-4 cells (**d**–**f**) examined by immunostaining against α-SMA. The specimens were counterstained with hematoxylin. Entire view of the model, with HSC-3 (**a**) and HSC-4 cells (**d**) seeded on top of the stromal layer. Two arrowheads indicate the borders between the “normal oral mucosa” and “oral cancer tissue” portions (**a**,**d**). Magnified microscopic images of the “normal oral mucosa” (**b**,**e**) and “oral cancer tissue” (**c**,**f**) portions are also displayed. Scale bar in (**a**,**d**): 2 mm. Scale bar in (**b**,**c**,**e**), and (**f**): 100 μm.

Finally, we examined the expression pattern of α-SMA and LRRC15, potential CAF markers, in our model. Most α-SMA-positive cells were localized in close proximity to the OSCC (HSC-3/HSC-4 cells) layer and the oral mucosa epithelial layer, differing from the distribution patterns of prelabeled PD-CAFs and PD-NOFs and the expression level in the 2D culture condition. This led to a specific feature wherein α-SMA-positive cells were unevenly distributed within the stromal layer (Figure 9a–f). The number of α-SMA-positive cells was remarkably higher in the cancer stroma (Figure 9c,f) than in the normal oral mucosa connective tissue (Figure 9b,e). Furthermore, a larger number of α-SMA-positive cells were found in the HSC-3 model than in the HSC-4 model (Figure 9c,f).

**Figure 10:**
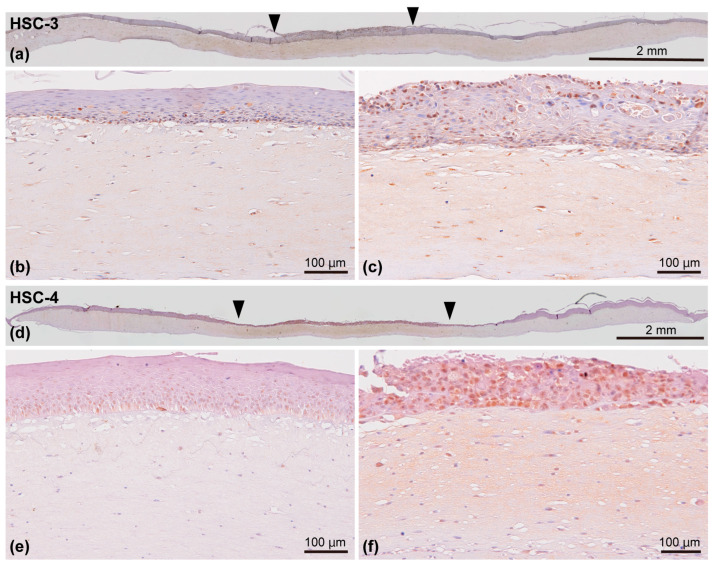
Representative immunohistochemical appearances of the 3D organotypic in vitro oral cancer model comprising HSC-3 (**a**–**c**) and HSC-4 cells (**d**–**f**) examined by immunostaining against LRRC15. The specimens were counterstained with hematoxylin. Entire view of the model with HSC-3 (**a**) and HSC-4 cells (**d**) seeded on top of the stromal layer. Two arrowheads indicate the borders between the “normal oral mucosa” and “oral cancer tissue” portions (**a**,**d**). Magnified microscopic images of the “normal oral mucosa” (**b**,**e**) and “oral cancer tissue” (**c**,**f**) portions are also presented. Scale bar in (**a**,**d**): 2 mm. Scale bar in (**b**,**c**,**e**), and (**f**): 100 μm.

Regarding LRRC15, the cancer stroma displayed a diffuse immunoreaction, whereas scarce immunoreaction was observed in the normal oral mucosa connective tissue (Figure 10a,d). Similarly, most PD-CAFs in the cancer stroma were positive (Figure 10c,f), while PD-NOFs were scarcely positive (Figure 10b,e). Additionally, the basal layer of the oral mucosa epithelial layer and the lower layer of the OSCC layer were immuno-positive for LRRC15 in this model (Figure 10b,c,e,f).

## 4. Discussion

According to the 2020 global cancer statistics, the number of new cancer cases and cancer-related deaths related to cancers developing in the lip and oral cavity were 377,713 and 177,757, respectively, making up 2.0% and 1.8% of 36 cancer types. Most oral cancers are SCCs [25]. Although the five-year survival rate of OSCC patients has improved in the last few decades, their prognosis has stagnated despite advancements in diagnostic technologies and therapeutic strategies [2,26]. A better understanding of the molecular and cellular insights of TME and physiologically relevant models of oral cancer for the testing of potential drug treatments are required to provide the best possible care for OSCC patients [15,27,28]. Although our ultimate goal is to develop a more biomimetic 3D OSCC model, the current 3D organotypic in vitro oral cancer model, including PD-CAFs, is an innovative tool for oral cancer translational research because it mimics the positioning and environment of in vivo oral cancer [29].

This study used a stainless-steel partition, a pivotal technique enabling the co-culture of four different cell types. Although the vertical integration of the stromal layer and the epithelial component was successful in our previous studies [19,20,21,22], this small device, placed at day 0 and day 2–6 during manufacturing, enabled the continuous horizontal integration of the oral mucosa epithelial and OSCC layers into the organotypic model, resulting in the co-culturing of four cell types. Additionally, the partition’s thinness was effective in avoiding the gap between the components seeded inside and outside after removing the device, displaying a similar architecture of oral cancer tissue in vivo vertically and horizontally. Different from the environment of standard organoid culture—because the epithelial/cancer cell layer was exposed to the air, similar to the oral cavity, and nourished only from the underlying stromal layer, this model is currently the most biomimetic 3D in vitro one recapitulating in vivo oral cancer tissue.

Another vital component of this organotypic oral cancer model is the repopulation of paired PD-CAFs and PD-NOFs in the stromal layer, leading to personalized medicine for oral cancer patients, as long as PD-oral squamous carcinoma cells and normal oral keratinocytes are replaced with the current cells of this study [13]. Here, PD-CAFs were isolated using our own technique and serially passaged to expand the cell population. CAFs are a heterogeneous cell population arising from various cell types, including resident fibroblasts, endothelial cells, mesenchymal stem cells, and epithelial cells undergoing epithelial–mesenchymal transition [30,31]. Although reports on specific CAF markers have been limited, α-SMA expression, exhibiting a myofibroblastic feature, has been generally considered a CAF marker [32,33]. Based on the differential α-SMA expression in the 2D culture condition, the fibroblastic cells obtained from the oral cancer tissue core, and grown and serially passaged in culture, were confirmed to be PD-CAFs, distinguishable from PD-NOFs. In contrast, we found a distinctive distribution pattern of α-SMA-positive PD-CAFs in the 3D model, although PKH-26 prelabeled cells were distributed in the entire cancer stromal layer. This characteristic finding of the remarkable accumulation of α-SMA-positive PD-CAFs in close proximity to the OSCC layer, similar to the OSSC surgical specimens (Supplementary Figure S3), suggests crosstalk and interactions between HSC-3/HSC-4 cells and PD-CAFs via direct cell contact or secreted molecules in this 3D organotypic model [34,35,36]. A similar localization pattern of α-SMA-expressed PD-NOFs was observed in the normal epithelial portion despite a much lower density. However, a previous study suggested that ductal myoepithelial cells expressing α-SMA in breast cancer could suppress cancer progression [37]. Additional examinations are necessary to unveil the molecular mechanism of TME in oral cancer.

Furthermore, the distribution of PD-CAFs and PD-NOFs prelabeled with PKH-26 and PKH-67, respectively, indicated that PD-CAFs did not migrate far across the boundary of two stroma portions during manufacturing and remained in the original portion seeded at the beginning. Although studies on various cancers indicated that tumor-specific CAFs promote cancer cell migration, few reports investigated the migrating ability of CAFs, even in a 2D cell culture. In non-small cell lung cancers, the integrin-α11 expression in CAFs via type I collagen production enhanced CAF migration, confirming that integrin-α11 expression can be a new CAF marker [38]. Because increased integrin-α11 expression in CAFs resulted in a poor prognosis of cancer patients, further analysis on the TME of oral cancer using this organotypic model must be conducted. Additionally, a distinctive and diffuse immunoreaction of LRRC15 was observed in the cancer stroma, suggesting that PD-CAFs and ECM are immuno-positive for LRRC15. This area was consistent with the zone where PD-CAFs were present, although the basal cells of the oral mucosa epithelial layer and the HSC-3/HSC-4 cells also expressed LRRC15. Since recent studies demonstrated that LRRC15 was expressed in stromal fibroblasts in many solid tumors (e.g., breast, head and neck, lung, or pancreatic), our findings suggest that LRRC15 can be used as a novel CAF marker [39,40]. Therefore, LRRC15 is likely to be a CAF marker of OSCC. The ECM immunoreaction with LRRC15 also indicated that this protein was soluble and released from cellular components, especially the “oral cancer tissue” portion [39,41,42].

Another characterization of this 3D organotypic model includes the notable expression pattern change of laminin-γ2 between the oral mucosa epithelial and OSCC layers. Laminin-γ2 is an essential component of the basement membrane of oral mucosa [43]. Previous studies highlighted that the immunohistochemical expression and distribution patterns of laminin-γ2 in the OSCC cancer tissue in vivo were correlated with the clinical prognosis of cancer patients [44,45,46]. More importantly, recent papers reviewed laminin-γ2 as a driver of OSCC development and progression because its lack of basement membrane structure and its ECM deposition are relevant to oncological events, including stromal activation and epithelial-to-mesenchymal transition, indicating that laminin-γ2 can be an attractive therapeutic target of OSCC [47,48]. Therefore, the loss of the basement membrane structure and diffuse expression within the cancer layer, where the extracellular laminin-γ2 deposition of HSC-3 was more diffuse and remarkable than in HSC-4 cells, present this model as a useful tool to investigate OSCC cellular responses in anti-cancer strategies.

The histopathological features of the OSCC (HSC-3/HSC-4 cells) layer revealed that the cells grew over the surrounding oral mucosa epithelial layer, resulting from HSC-3 and HSC-4 cells crawling up over that layer at the border area. Although the length of the outward growth of HSC-3/HSC-4 cells varied among the 98 models manufactured, the phenomenon could be due to the higher proliferating capacity of HSC-3/HSC-4 cells than NOKs, supported by the drastic difference in the distribution pattern of Ki-67-positive cells. Nonetheless, the lateral invasion of HSC-3/HSC-4 cells has drawn special attention because it leads to poor survival [49]. The “lateral invasion” is determined by the spread of small cancer cell foci to the submucosa under the normal epithelium [49]. Although the border between the HSC-3/HSC-4 cells and NOKs was relatively clear in our histopathological examination, the localization of Ki-67-positive cells at the boundary between the OSCC layer and the oral mucosa epithelial layer may display the invasion of small HSC-3/HSC-4 cell clusters. Therefore, this model may be applied to study “lateral invasion” in vitro. Moreover, this model can be used to further elucidate the communication between cancer cells and normal epithelial cells because HSC-3/HSC-4 cells have direct contact with NOKs.

In contrast, the invasive behavior of HSC-3/HSC-4 cells in the stromal layer, especially HSC-3 cells, was not obvious in this study, differing from our previous study [19]. This discrepancy could be due to a relatively shorter period of co-culturing with the underlying stromal layer (9 days vs. 14 days). The previous study, generating a different “organotypic oral cancer model”, demonstrated that HSC-3 cells deeply invaded the human leiomyoma tissue [50,51]. The study concluded that myoma is a useful scaffold to study the tumor’s invasive behavior because it contains ECM, such as laminin-γ2, mimicking the TME, and induces the gelatinase and collagenase production from HSC-3 cells. Co-culturing for a longer period may be required to investigate the invasive behavior of OSCC cells in this model. Furthermore, it is necessary to further characterize the model’s stromal layer, including ECM molecules, proteases, and the epithelial–mesenchymal transition [50,51].

Although the current form is a prototype of a biomimetic 3D oral cancer model, it displays several advantages. First, it presents an alternative to animal testing. Second, it allows us to investigate the cellular responses of HSC-3/HSC-4 cells and oral mucosa epithelial cells simultaneously and in the same model. For example, the specific dose of anti-cancer agents can be measured and determined by cellular responses. Additionally, similarly to our previous study [22], this model can be used to analyze and evaluate the effects of radiation therapies on cancers because irradiation treats malignancies locally. Therefore, the 3D organotypic model is vital as a preclinical model. Third, depending on the size of the culture insert and the partition, the model’s size is changeable. Fourth, the model allows easy access to the apical side of the epithelial component because of the biomimetic polarity. Lastly, owing to the co-culturing of four cell types, a novel research strategy of spatial-temporal cancer biology can be applied [5,52]. The applicability and feasibility of this 3D organotypic model are expected to increase for various oral cancer studies.

Nevertheless, this model presents basic disadvantages due to the manual workflow, like any organotypic culture model, including the vital explant co-culture model [53]. It includes low throughput, a lack of reproducibility, limited scalability, and skillful handling. Moreover, this model does not self-assemble and self-organize and cannot be cryopreserved. Due to the co-culture system, analyzing the culture supernatant is impossible. Additionally, live imaging is undoable, differing from standard organoid culture. Since different assessments require different culture model formats and specific features, it is appropriate to use our organotypic and organoid models properly and separately, depending on individual cancer research interests [14]. Our final goal is to incorporate blood vessels (endothelial cells) and immune cells into this model to increase the values of utility and enhance the quality of preclinical testing as an evaluation tool [11,28,54]. Such incorporation of specific cellular elements serves as the third TME component and can provide a more biomimetic 3D oral cancer model [55]. Comprehensive work using reliable 3D cell culture models should contribute to predicting the therapy outcomes of oral cancer patients and advance precision therapies [13,56].

The current format of this organotypic oral cancer model can be used as a tool for screening and developing new anti-cancer drugs, particularly those targeting laminin-γ2 deposition in the OSCC layer. Additionally, given the increase in advanced oral cancer cases, this model is useful for evaluating the effects of multidisciplinary therapy that combines chemotherapy and radiotherapy, not only on the OSCC layer but also on the responses of normal oral mucosa. Furthermore, because this biomimetic oral cancer model can simulate the TME of oral cancer, the epithelial–mesenchymal transition in tumor tissues and the interactions of OSCC with ECM and neighboring stromal and normal cells will also be investigated.

## 5. Conclusions

This study was successful in developing a 3D organotypic in vitro oral cancer model by co-culturing PD-CAFs, PD-NOFs, NOKs, and HSC-3/HSC-4 cells. This model’s basic structure comprised the bi-layered oral cancer tissue at the center and the surrounding normal oral mucosa. This vertical and horizontal positioning with four distinct and adjoining portions displayed polarity and mimicked oral cancer developing in the oral mucosa. Histopathological findings demonstrated that α-SMA-positive PD-CAFs in the cancer stroma were localized in close proximity to the OSCC layer, suggesting a crosstalk between HSC-3/HSC-4 cells and CAFs. Furthermore, laminin-γ2 was not localized at the interface between the OSCC layer and the underlying cancer stroma but diffusely present within the entire OSCC layer, indicating the loss of the basement membrane-like structure in the cancer tissue. The 3D organotypic in vitro oral cancer model presented great potential to further understand oral cancer biology in vitro, especially TME as one of the major therapeutic targets.

## 6. Patents

This work has been submitted for a patent application to the Patent Office of Japan. (Patent application number: 2024-040964; 15 March 2024).

## Data Availability

The data that support the findings of this study are available from the corresponding author on reasonable request.

## Supplementary Materials

The following supporting information can be downloaded at https://www.mdpi.com/article/10.3390/biomedicines12102373/s1. Figure S1. Illustrations of the area where the tissue samples were obtained. (a) Primary oral cancer diagnosed with squamous cell carcinoma of the tongue. (b) Resected surgical specimen. The specimen was cut and examined along the white dotted line. (c) The white boxes indicate the portions from which the sampling tissue was obtained for CAFs and NOFs. Figure S2. Illustrations of the procedure to establish primary PD-CAF cell culture. (a) The cancer specimen was grated onto a 60 mm dish, whose surface was finely scratched. (b) Before the surface dried completely, DMEM with 10% FBS supplemented with gentamicin and amphotericin B was gently added. Figure S3. Representative images of the surgical specimens stained with H&E (a) and immunostained with α-SMA (b). (c,d) are magnified from the black box in the lower power image of (a) and (b), respectively. Scale bars = 100 μm. Figure S4. Representative immunohistochemical image of the human gingiva sample in vivo. The specimen was examined using immunofluorescent staining against laminin-γ2 (green), integrin-α6 (red), and DAPI (blue). Co-expression of laminin-γ2 and integrin-α6 was seen at the basement membrane zone in the merged image. Scale bars = 100 μm. File S1. Mycoplasma test reports for HSC-3 and HSC-4 cells. File S2. Cell authentication report of HSC-3 and HSC-4 cells from JCRB Cell Bank.
