# Supplementary material for: Development and Characterization of a Three-Dimensional Organotypic In Vitro Oral Cancer Model with Four Co-Cultured Cell Types, Including Patient-Derived Cancer-Associated Fibroblasts
Source: Biomedicines. 2024 Oct 17;12(10):2373. doi: 10.3390/biomedicines12102373 (PMC11505046; doi:10.3390/biomedicines12102373)
Supplement: Supplementary file 1 [file biomedicines-12-02373-s001.zip › File S1.pdf]

Test Report

Basic information

Requester: Funakoshi Co., Ltd.

Facility: Division of Biomimetics, Faculty of Dentistry and Graduate School of Medical and Dental Sciences, Niigata University

Date of sampling: –

Date of sample recieving: 11-Jun-24

Date of report: 12-Jun-24

Test results

ID :	Sample	Categories	Results	
K12409	HSC-3 culture supernatant	■ Mycoplasma Test	Negative (—)	Real-time PCR
K12410	HSC-4 culture supernatant	■ Mycoplasma Test	Negative (—)	Real-time PCR

Notes

Biotherapy Institute of Japan, Inc.

Domestic call: 03-5632-6080 International call: +81-3-5632-6080
