# Supplementary material for: Development and Characterization of a Three-Dimensional Organotypic In Vitro Oral Cancer Model with Four Co-Cultured Cell Types, Including Patient-Derived Cancer-Associated Fibroblasts
Source: Biomedicines. 2024 Oct 17;12(10):2373. doi: 10.3390/biomedicines12102373 (PMC11505046; doi:10.3390/biomedicines12102373)
Supplement: Supplementary file 1 [file biomedicines-12-02373-s001.zip › File S2.pdf]

19/June/2024

JCRB Cell Bank

National Institute of Biomedical Innovation
7-6-8 Saito-Asagi, Ibaraki, Osaka, Japan
TEL : +81-72-641-9851
FAX : +81-72-641-9859

Cell Authentication Report (KBN0956)

Client:

Kenji Izumi
Niigata University, Faculty of Dentistry, Div of Biomimetics
TEL : 025-227-2850
E-mail : kenniea22002@yahoo.co.jp

Sample Information

Sample Number : 2

1) Cell No. : KBN0956-01 (Cell Name : HSC-3)
2) Cell No. : KBN0956-02 (Cell Name : HSC-4)

Peak report (Cell No. : KBN0956-01)

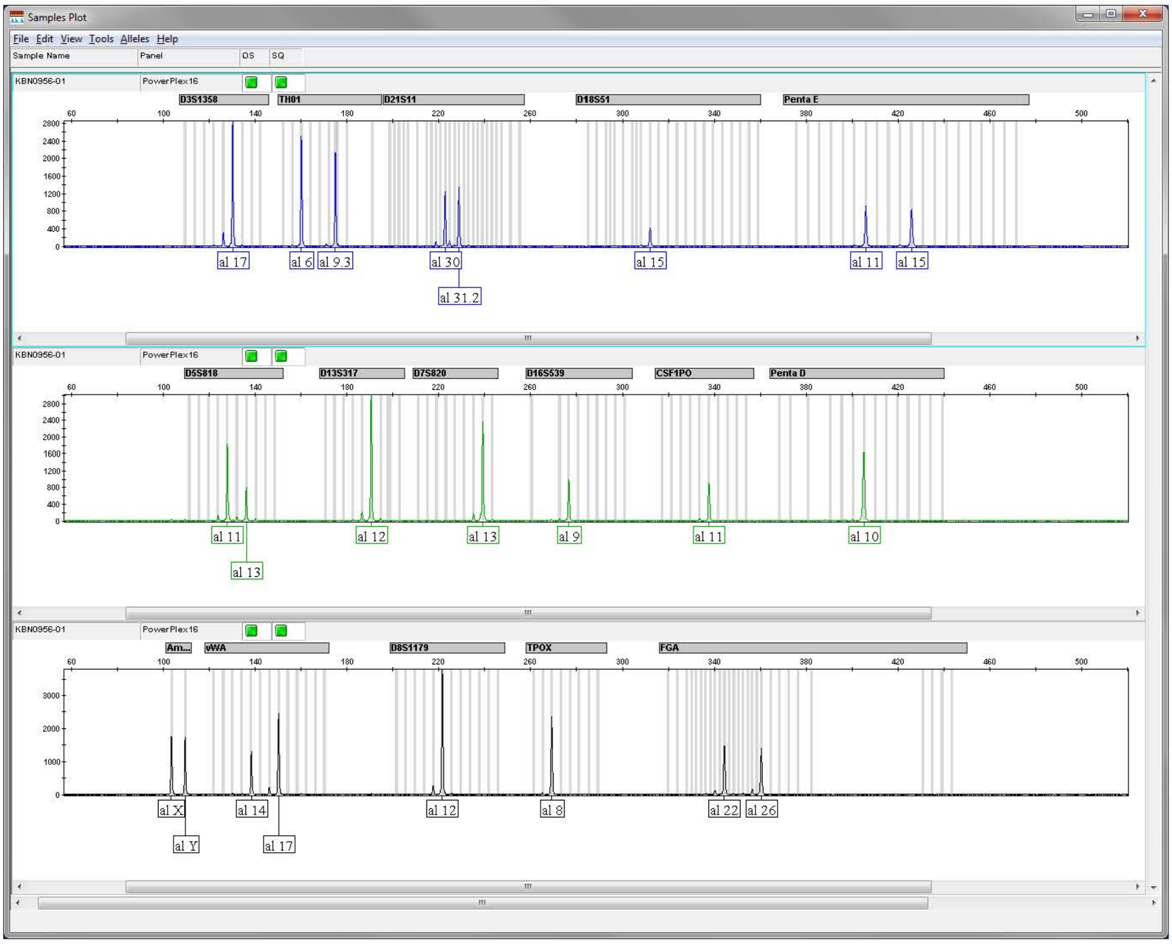

STR Profile (Cell No. : KBN0956-01)

D3S1358	TH01	D21S11	D18S51	Penta_E
17	6,9.3	30,31.2	15	11,15

D5S818	D13S317	D7S820	D16S539	CSF1PO	Penta_D
11,13	12	13	9	11	10

AM	vWA	D8S1179	TPOX	FGA
X,Y	14,17	12	8	22,26

Comparison with database (Cell No. : KBN0956-01)

Cell No.	Cell Name	EV	D5S818	D13S317	D7S820	D16S539	VWA	TH01	AM	TPOX	CSF1PO
**KBN0956-01**	**HSC-3**	**----**	**11,13**	**12**	**13**	**9**	**14,17**	**6,9.3**	**X,Y**	**8**	**11**
JCRB0623	HSC-3	**1.000**	11,13	12	13	9	14,17	6,9.3	X,Y	8	11
JCRB1354	HSC-3-M3	**1.000**	11,13	12	13	9	14,17	6,9.3	X,Y	8	11
RCB1975	HSC-3	**1.000**	11,13	12	13	9	14,17	6,9.3	X,Y	8	11
JCRB1685	HSC-3 CMV-Luc	**1.000**	11,13	12	13	9	14,17	6,9.3	X,Y	8	11
RCB1893	TE-7	0.667	11	12	10,13	9,10	17	9	X,Y	8	11
RCB1948	TE-2	0.667	11	12	10,13	9,10	17	9	X,Y	8	11
JCRB0199	huH-1	0.611	10,13	10,12	11	9	17,18	6,9	X,Y	8,11	11
JCRB1384	STR-428	0.611	10,13	11,12	11	9,12	14	6,9	X,Y	8	11
ACC-374	KYSE-510	0.611	11	12	11,12	9	14	9	X	8	11
CRL-4011	NuLi-1	0.611	11,13	10,12	12,13	8,13	14,18	9.3	X,Y	8,11	11

Summary (Cell No. : KBN0956-01)

It was confirmed that the cell (Cell No. : KBN0956-01, Cell Name : HSC-3) was the same as the cell registered in JCRB Cell Bank (JCRB0623 HSC-3, JCRB1354 HSC-3-M3, JCRB1685 HSC-3 CMV-Luc), and the cell registered in RIKEN BRC Cell Bank (RCB1975 HSC-3), by the comparison with the database of JCRB Cell Bank.

Peak report (Cell No. : KBN0956-02)

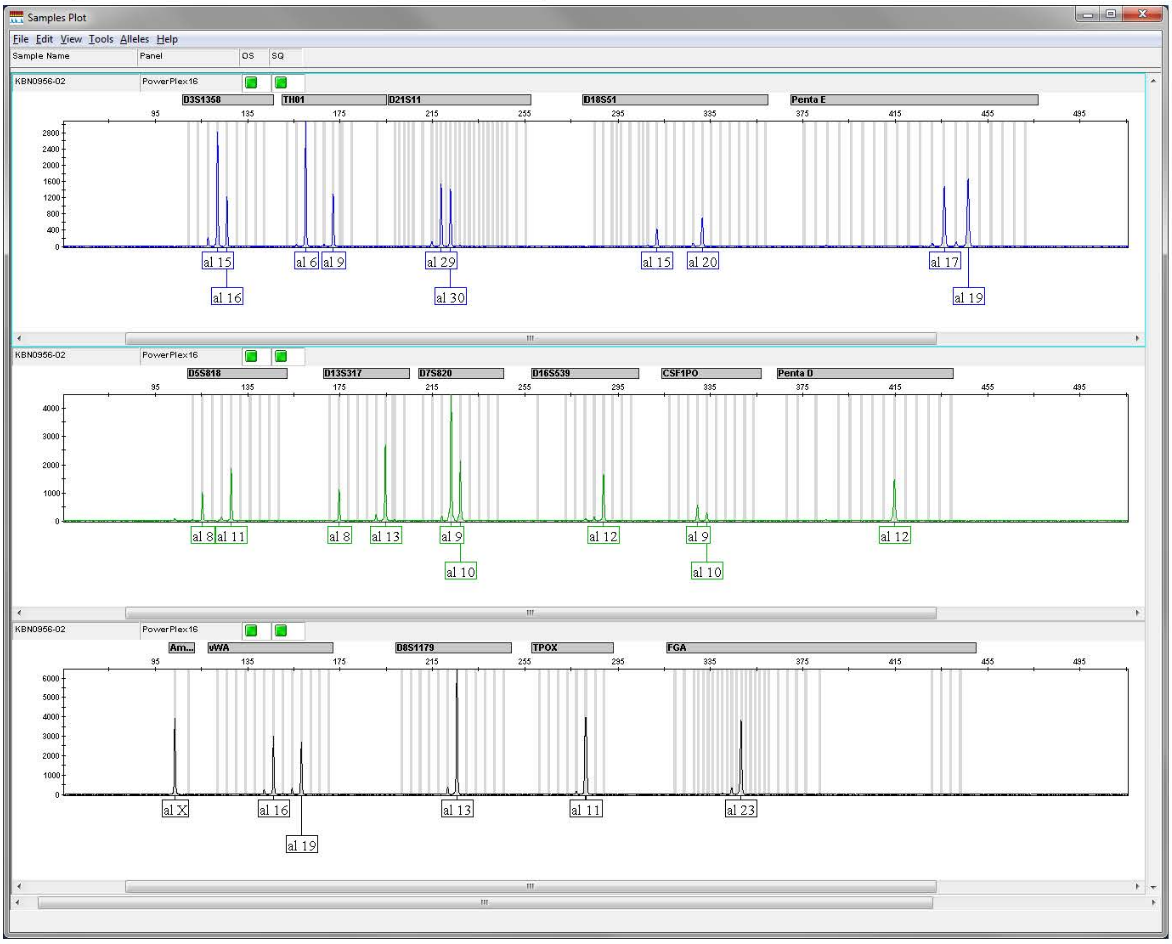

STR Profile (Cell No. : KBN0956-02)

D3S1358	TH01	D21S11	D18S51	Penta_E
15,16	6,9	29,30	15,20	17,19

D5S818	D13S317	D7S820	D16S539	CSF1PO	Penta_D
8,11	8,13	9,10	12	9,10	12

AM	vWA	D8S1179	TPOX	FGA
X	16,19	13	11	23

Comparison with database (Cell No. : KBN0956-02)

Cell No.	Cell Name	EV	D5S818	D13S317	D7S820	D16S539	VWA	TH01	AM	TPOX	CSF1PO
**KBN0956-02**	**HSC-4**	**----**	**8,11**	**8,13**	**9,10**	**12**	**16,19**	**6,9**	**X**	**11**	**9,10**
RCB1902	HSC-4	**1.000**	8,11	8,13	9,10	12	16,19	6,9	X	11	9,10
JCRB0624	HSC-4	**0.944**	8,11	8,13	9,10	12	16,19	6	X	11	9,10
TKG 0489	HSC-4	**0.944**	8,11	8,13	9,10	12	16,19	6,9	X	11	9
RCB0629	SF8419	0.722	11,12	8	10,12	12	16,19	6,9	X	8,11	10,12
JCRB0826	8505C	0.667	10,11	13	10	12	17,19	6,9	X	11	12,13
ACC-693	ELF-153	0.667	11	8	9,10	12,13	19	6,9	X	8,11	10
ACC-665	UPCI-SCC-099	0.632	11,12	8,13	7,9,10	11,12	14,18,19	6,9.3	X	11	11,12
RCB0628	SF8420	0.611	10,11	8,9	9,11	9,10	16,19	9	X	11	10
RCB2103	8505C	0.611	10,11	13	10	12	17,19	6,9	X	10,11	12,13
ACC-38	PF-382	0.611	11,12	11,12	9,10	9,12	16,17	6,9.3	X	11	10,13

Summary (Cell No. : KBN0956-02)

It was confirmed that the cell (Cell No. : KBN0956-02, Cell Name : HSC-4) was the same as the cell registered in RIKEN BRC Cell Bank (RCB1902 HSC-4), the cell registered in JCRB Cell Bank (JCRB0624 HSC-4), and the cell registered in Tohoku University Cell Resource Center for Biomedical Research Cell Bank (TKG 0489 HSC-4), by the comparison with the database of JCRB Cell Bank.
