# Supplementary figures and images for: Development and Characterization of a Three-Dimensional Organotypic In Vitro Oral Cancer Model with Four Co-Cultured Cell Types, Including Patient-Derived Cancer-Associated Fibroblasts
Source: Biomedicines. 2024 Oct 17;12(10):2373. doi: 10.3390/biomedicines12102373 (PMC11505046; doi:10.3390/biomedicines12102373)

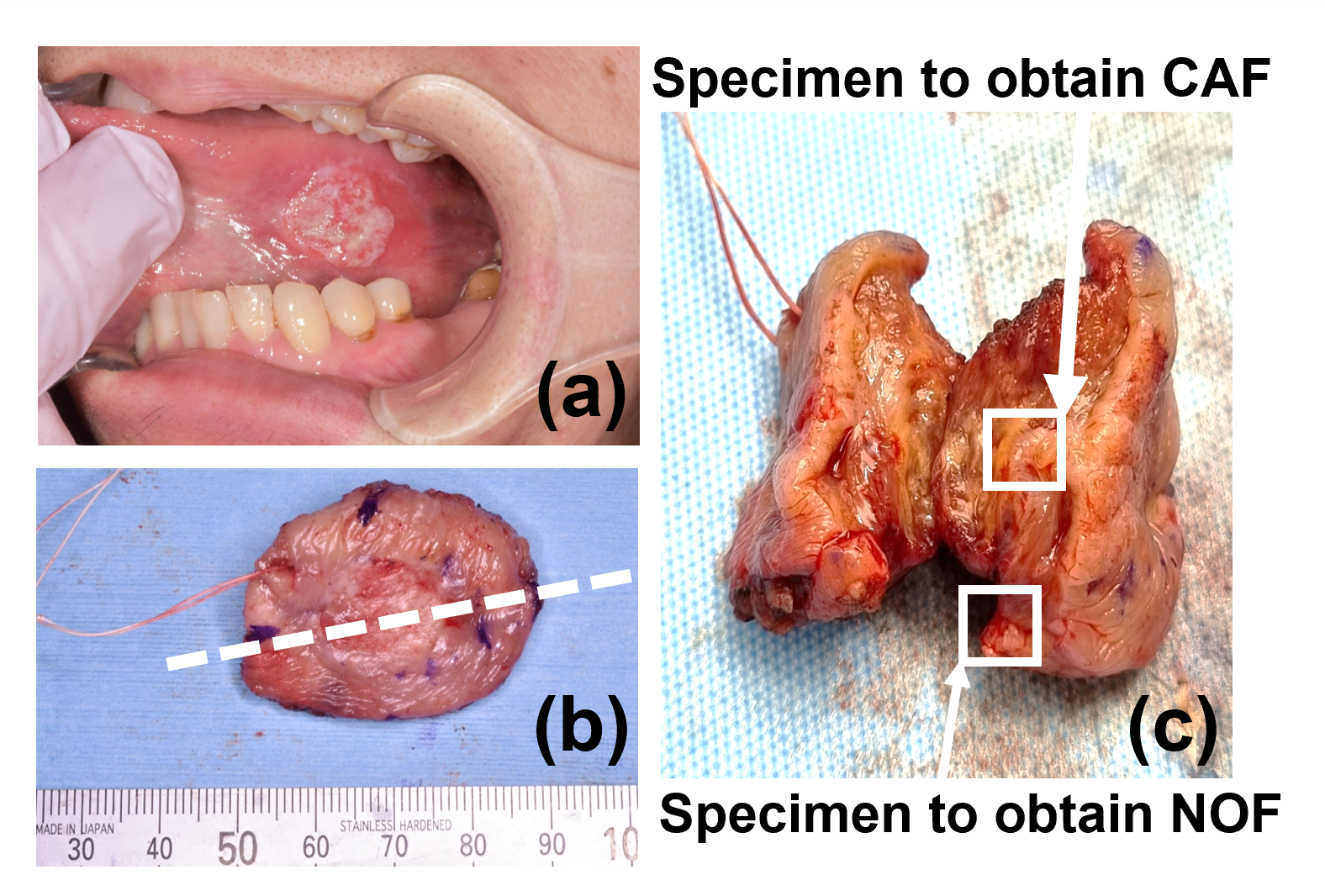

Supplement: Supplementary file 1 [file biomedicines-12-02373-s001.zip › Figure S1.tif]

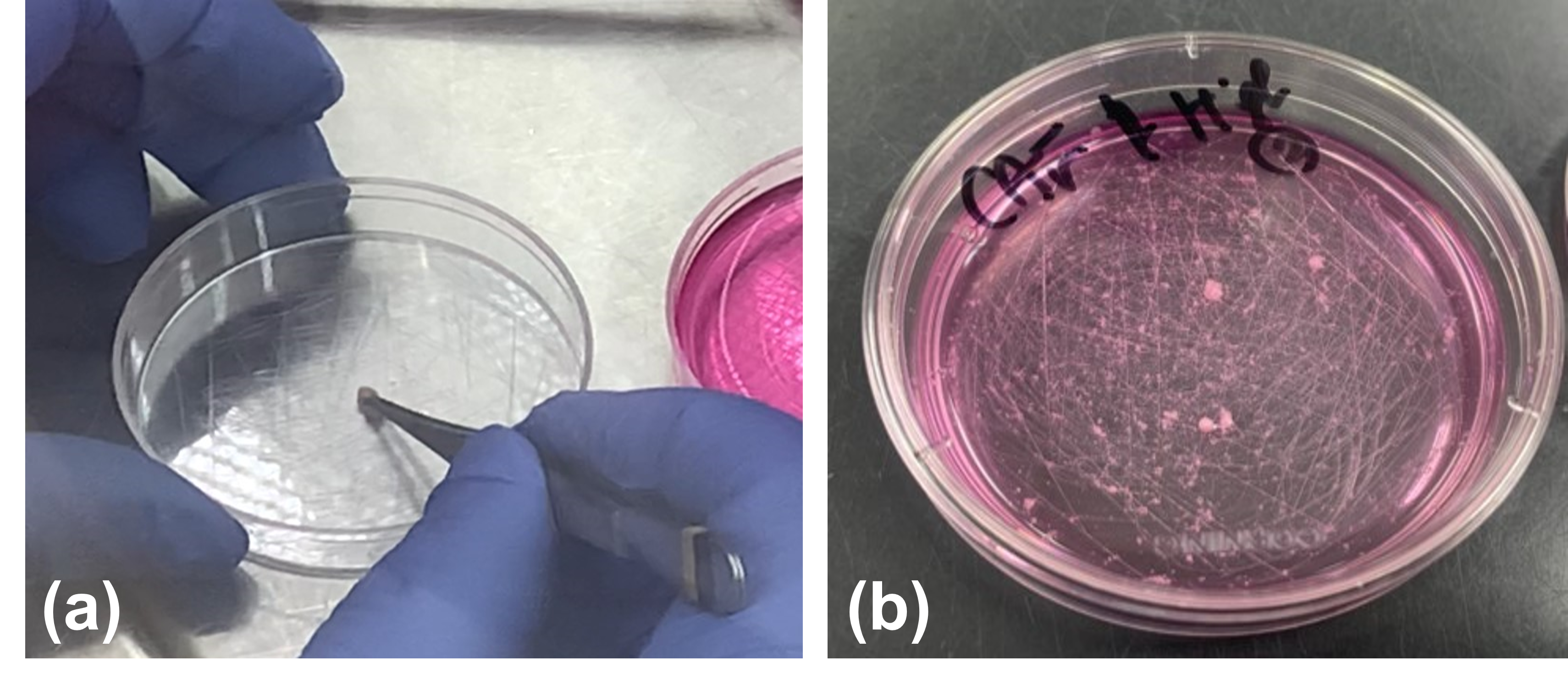

Supplement: Supplementary file 1 [file biomedicines-12-02373-s001.zip › Figure S2.tif]

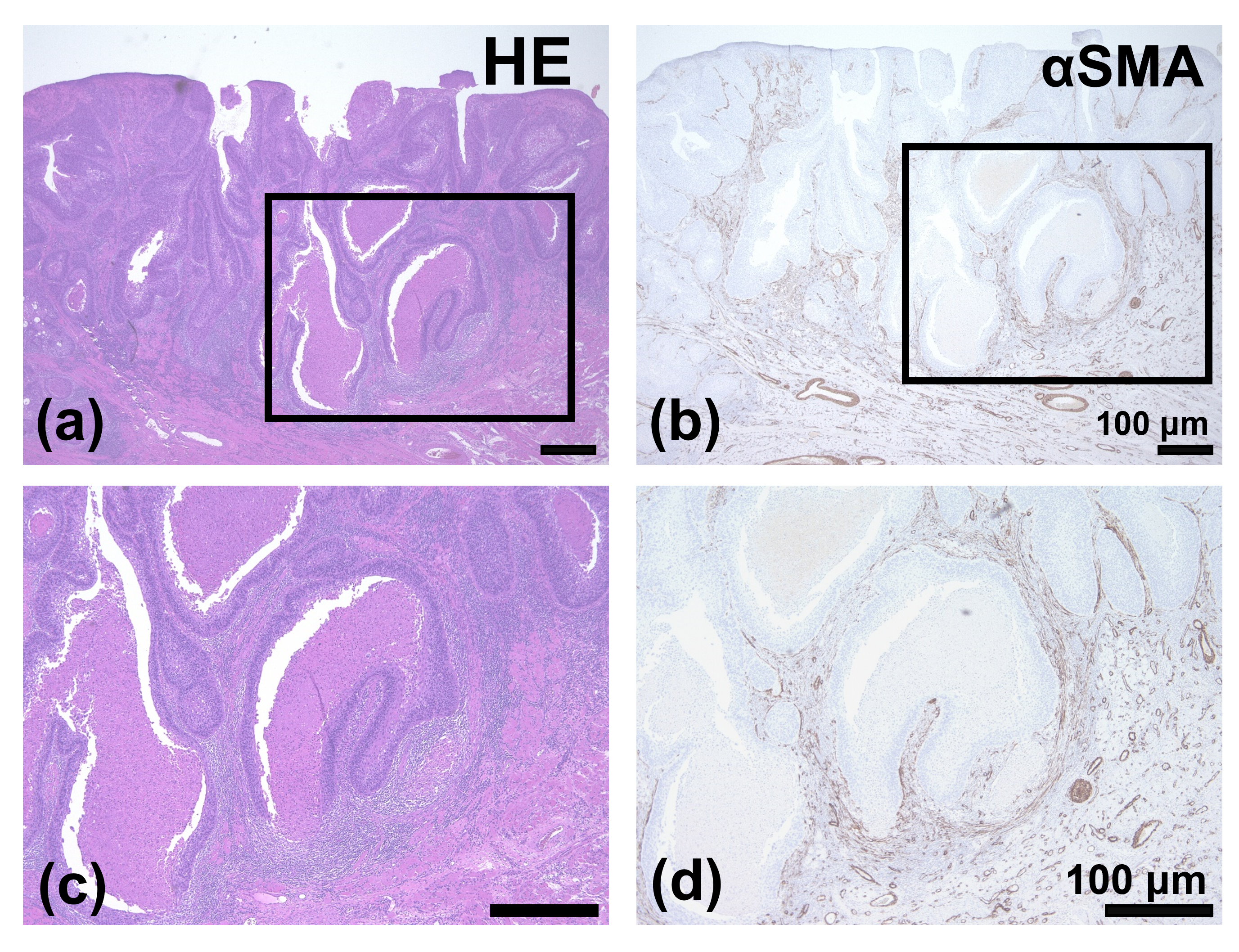

Supplement: Supplementary file 1 [file biomedicines-12-02373-s001.zip › Figure S3.tif]

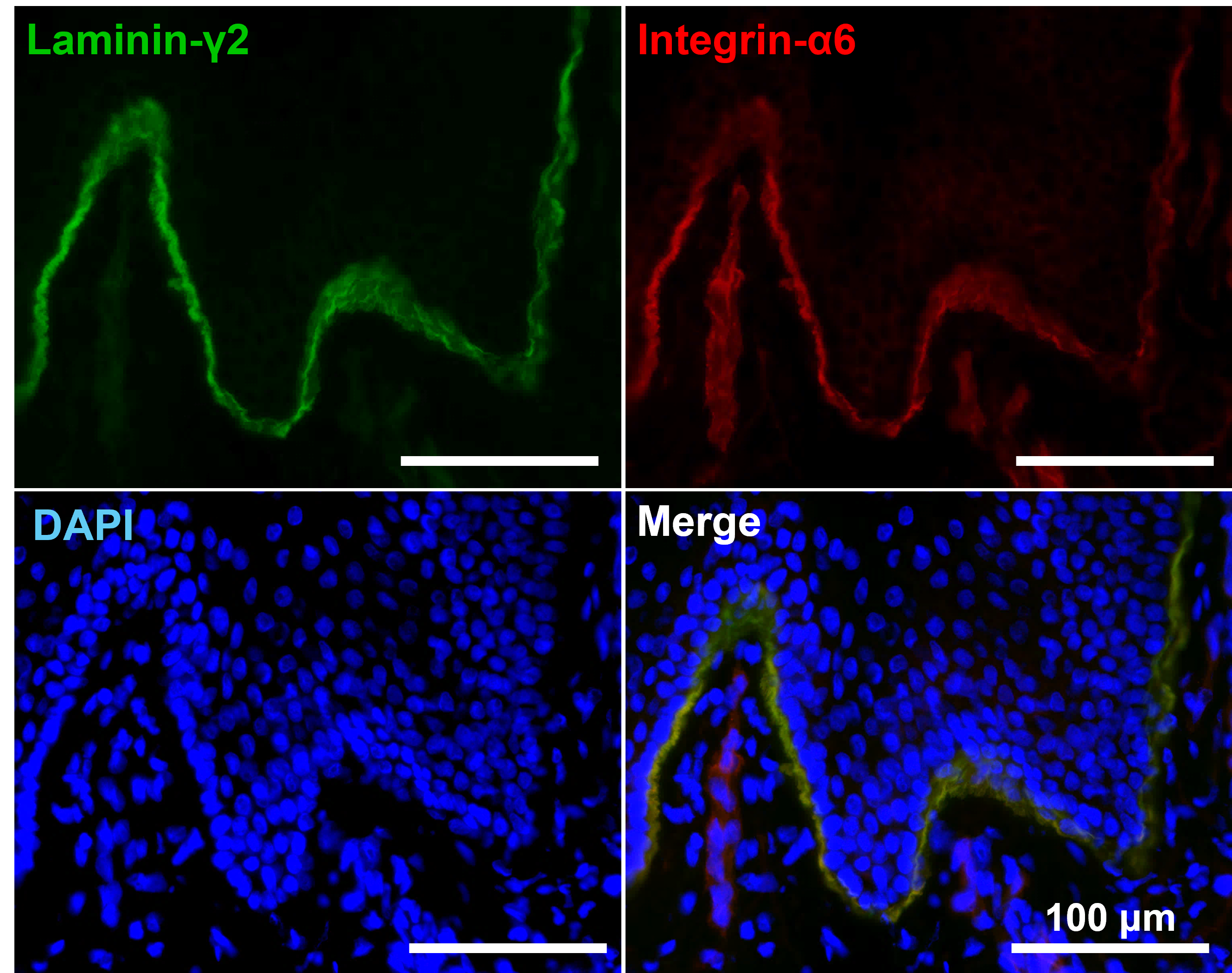

Supplement: Supplementary file 1 [file biomedicines-12-02373-s001.zip › Figure S4.tif]
